# Microbiological quality assessment (including antibiogram and threat assessment) of bottled water

**DOI:** 10.1002/fsn3.2164

**Published:** 2021-02-06

**Authors:** Bikram Gautam

**Affiliations:** ^1^ Research Center for Applied Science and Technology (RECAST) Tribhuvan University Kritipur Nepal; ^2^ Department of Microbiology St. Xavier's College Kathmandu Nepal

**Keywords:** bottled water, bacteriophage, coliform, multi drug resistant bacteria, parasite, *Pseudomonas*, threat assessment

## Abstract

Water pollution is a major global problem that has been the leading cause of morbidity and mortality. This study was carried out to assess the microbiological quality of popular domestic brands of bottled water available in the Kathmandu Valley in Nepal. For the study of bottled water, a total of 50 samples each of different volumes (20 and 1 L) were selected. The samples were processed at the Microbiology laboratory at St. Xavier's College, Maitighar, Kathmandu. The microbiological assessments were performed as per the methods described in the American Public Health Association, 2005. Out of 100 samples, 48% of samples were found to be contaminated with total coliform. *Escherichia coli* was the predominant strain among the coliforms. Multidrug‐resistant *E. coli*, *Enterobacter aerogenes*, and *Pseudomonas aeruginosa* were isolated from the 48 bottled water. Treat assessment test revealed that 88.23% of the isolated *E. coli* produced β hemolytic colonies, while 11.77% did not show hemolytic colonies, 100% *E. aerogenes* colonies were not able to develop hemolytic colonies and 100%, *P. aeruginosa* colonies gave β hemolytic colonies, respectively. Chi–square test shows that there is a significant association (*p* ≤ .05) between fecal coliform and volume of the vessel (i.e., 20 and 1 L), bottle type (i.e., unscratched and undented and scratched and/or dented), season (i.e., monsoon and postmonsoon), and total coliform. Chi‐square test shows that there is no significant (*p* > .05) association between *Pseudomonas* spp and season (i.e., monsoon and postmonsoon). Out of 100 samples, 48% of samples were found to be contaminated with total coliform. Microorganisms survive in bottled water as they have many nutrients required for the microorganism in ionic form. Surveillance is lacking by the license‐providing organizations followed by governmental organizations.

## Graphical abstract

Due to microbial contamination in bottled water, quality of the same has been decreasing across the globe (including developed countries). After assessing microbiological parameters of 100 samples, our study revealed that season and physical structure of bottle type also aid in the proliferation of microbial contamination thanks to their various survival mechanisms like biofilms, multi drug resistant patterns, survival in the low nutrient environment, horizontal and vertical mutations etc. This study also presents the threat assessment of the isolated bacteria when it is able to evade our immune system.

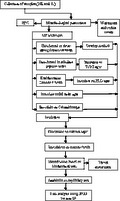

## 1 INTRODUCTION

Water pollution is a major global problem which has been the leading cause of morbidity and mortality worldwide and requires evaluation and revision of bottled water policy (Gautam et al., 2021). Almost 90% of child deaths are due to dehydration resulting from diarrheal disease as the sporadic, endemic, and epidemic cases of diarrhea are responsible for the deaths of around 1.5 million people every year (WHO, 2017).

The failure to provide safe drinking water and adequate sanitation services to all people is the most challenging aspect of the 21st century (Gautam, 2020; Gautam & Adhikari, 2018b; Gautam et al., 2021). Water‐related diseases have nearly been eliminated in developed countries, but they remain an alarming concern in developing countries (Gautam, 2020; Gautam & Adhikari, 2018b; Gautam, Aryal, et al., 2018; Gautam et al., 2021; WHO, 2017). An outbreak may occur when fecal contaminants enter the water supply, as pathogens (such as *Salmonella* spp, *Shigella* spp, *Vibrio cholerae*, and *Escherichia coli*) may be shed in human feces (Gautam & Adhikari, 2018a, 2018b; Gautam, Aryal, et al., 2018; Gautam et al., 2019, 2021). Like many cases, a significant proportion of water‐borne illnesses are likely to go unnoticed by the communicable diseases surveillance reporting systems (Bonita et al., 2006). Since many illnesses are undiagnosed and unreported, the true extent of these diseases are still unknown (WHO, 2017).

Due to increased demand and consumption of bottled water, there has been a growing concern about the quality of these products (Gautam, 2020; Gautam & Adhikari, 2018b; Gautam et al., 2021). Bottled water quality can be deteriorated by microbial and toxic chemicals (Gautam, 2020; Gautam et al., 2021). The presence of contaminants in bottled water can lead to adverse health effects including gastrointestinal illness, reproductive problems, and neurological disorders (Gautam, 2020; Gautam & Adhikari, 2018a; Gautam, Aryal, et al., 2018; Gautam et al., 2021; Gautam, Rajbhanshi, et al., 2019). Bottled water can be the source of the causative agent invoking cholera, typhoid fever outbreaks, traveler's disease etc (Gautam, Aryal, et al., 2018; Gautam et al., 2021; Gautam, Rajbhanshi, et al., 2019; WHO, 2017) as contaminated water is a well‐known vehicle that leads to endemic, epidemic outbreaks (Bonita et al., 2006; Gautam, Aryal, et al., 2018; Gautam et al., 2021; Gautam, Rajbhanshi, et al., 2019; WHO, 2017).

In a study conducted by Atienza et al. (2017) in the Philippines, 88.89% of bottled water was contaminated with nonfecal coliform. 44.44% of bottled water samples exceeded the criteria of zero fecal *E. coli* coliform per 100 ml of drinking water.

The outcomes of this study are expected to enlighten the public on the importance of water quality. It also hopes to shed some light on the governmental bodies, water bottle industries, and license‐providing authoritative bodies like the Food and Drug Administration (FDA), International Bottled Water Association (IBWA), and Department of Food Technology and Quality Control (DFTQC). The results of this study hopes to help the bottled water industries improve their products to lower morbidity and mortality.

This study aims to assess the microbiological properties of bottled water by comparing them with the IBWA guidelines.

## 2 METHODOLOGY

### 2.1 Study site

The study was performed in the Department of Microbiology, St. Xavier's College, Maitighar.

### 2.2 Sample selection and size

For the bottled water study, both 20 and 1 L bottles were selected and processed in both monsoon and postmonsoon seasons. A total of 100 samples were selected for the study.

### 2.3 Sample collection, transportation, and storage

Using the aseptic technique (Gautam & Adhikari, 2018b; Gautam et al., 2021), the sample was drawn in four sterilized polypropylene bottles (Gautam, 2020), headspace was left and was labeled. The collected samples were kept in a minicooler with ice packs and transported to the microbiology laboratory to be processed within 4 hr. While the microbial assessment was being performed the remaining bottles were stored in a refrigerator (4°C).

### 2.4 Parasite(s)

One hundred milliliter bottled water was added in a sterile test tube and was centrifuged at 3,075 *g* for 20 min. Then, the water was decanted and the pellet portion was subjected to wet mount and iodine mount microscopy (APHA, 2005).

### 2.5 Heterotrophic plate count

Using a sterile micropipette, 1 ml of water samples was aseptically dispensed into sterile petri plates. Then, molted heterotrophic plate count (HPC) agar (≈45°C) was poured and moved in the clockwise and anticlockwise direction “8.” The plates were incubated for 24 hr at 37°C. The colony count was done after 24 hr. The result was expressed as cfu/ml of water (APHA, 2005; Gautam et al., 2021; Gautam, Rajbhanshi, et al., 2019). The formula for calculation is:

$$\text{Cfu}/\text{ml}\;=\;\frac{C\times \;D}{v}$$

where, *C* = total colonies counted; *D* = dilution fold; *v* = volume of sample taken.

### 2.6 Bacteria

#### 2.6.1 Coliform

One hundred milliliter of water was filtered through membrane filter (0.45 µm) apparatus. Membrane filter was inoculated in M‐endo agar plates and was incubated for 24 hr at 37°C (Gautam, Dongol, et al., 2017; Gautam, Pokhrel, et al., 2017). Same process was repeated and the membrane filter was subjected in M‐endo agar and was incubated at 44.5°C for 24 hr (Gautam, Aryal, et al., 2018). The result was expressed as number of cfu/100 ml of water (APHA, 2005; Gautam et al., 2021; Gautam, Rajbhanshi, et al., 2019). The isolates were subjected to biochemical tests. The formula for calculation is:

$$\text{Cfu}/100\;\text{ml}\;=\;\frac{C}{100}$$

where, *C* = total colonies counted.

#### 2.6.2 *Pseudomonas* spp

Measured volume of 100 ml water was filtered through membrane filter apparatus. Membrane filter was inoculated in cetrimide agar for 24 hr at 37°C. Same process was repeated and the membrane filter was subjected in cetrimide agar and was incubated at 42°C for 24 hr (Gautam, Aryal, et al., 2018). The result was expressed as number of cfu/100 ml of water (APHA, 2005; Gautam et al., 2021; Gautam, Rajbhanshi, et al., 2019). The isolates were subjected to biochemical tests. The formula for calculation is:

$$\text{Cfu}/100\;\text{ml}\;=\;\frac{C}{100}$$

where, *C* = total colonies counted.

#### 2.6.3 *Vibrio* spp

One hundred milliliter of water was filtered through membrane filter apparatus. Membrane filter was inoculated in 100 ml of alkaline peptone water of pH 8.6, for 6 hr at 35°C. Then, two loopful of broth was streaked on thiosulfate‐citrate‐bile‐sucrose agar and was incubated for 24 hr at 37°C (Gautam, Aryal, et al., 2018; Gautam, Rajbhanshi, et al., 2019). The isolates were subjected to biochemical tests.

#### 2.6.4 *Salmonella* spp

One hundred milliliter of water was filtered through membrane filter apparatus. Membrane filter was inoculated in 100 ml of Selenite F broth for 7 hr at 37°C. Two loopfuls of broth was streaked on Salmonella‐Shigella agar and was incubated for 24 hr at 37°C (APHA, 2005; Gautam, Aryal, et al., 2018; Gautam et al., 2021; Gautam, Rajbhanshi, et al., 2019). The isolates were subjected to biochemical tests.

### 2.7 Enrichment, isolation of coliphage

The isolated *E. coli* was inoculated in a sterile tube containing 5 ml nutrient broth. The tube was incubated for 4 hr at 37°C. Then, 5 ml of the pre‐incubated bacterial inoculum, 5 ml deca‐strength (10×) nutrient broth, 50 ml filtered water was mixed and was incubated for 24 hr at 37°C in a rotary shaker. Among the contents, 10 ml was filtered by the membrane filtration technique (filtrate was taken as enriched phage preparation). Using a sterile cotton swab, bacterial culture (tallied with McFarland standard 0.5) was transferred via swabbing in a nutrient agar plate. One hundred microliter of enriched phage preparation was added to the plate and incubated at 37°C for 24 hr (Gautam, Aryal, et al., 2018).

As a control, the same methodology was followed for *E. coli* (ATCC 25922) and *E. coli* (isolated from the sewage treatment plant, Guheswori).

The following day, pfu was observed (Gautam, Aryal, et al., 2018). The formula for calculation is

$$\text{Cfu}/\text{ml}\;=\;\frac{C\times \;D}{v}$$

where, *C* = total colonies counted; *D* = dilution fold; *v* = volume of the sample taken.

### 2.8 Antibiotic susceptibility test

The isolated organisms (turbidity tallied with McFarland standard 0.5) were subjected to antibiotic (of HiMmedia) susceptibility test according to Clinical and Laboratory Standards Institute (CLSI) guidelines (2016). As a control, antibiotic susceptibility test of *E. coli* (ATCC 25922) and *Pseudomonas aeruginosa* (ATCC 27853) bacteria was performed as quality control of the antibiotics (CLSI, 2016; Gautam, Nepal, et al., 2019).

### 2.9 Threat assessment

The isolated organism was streaked on the blood agar. The plates were incubated for 24 hr at 37°C. Hemolysis (α, β, γ) was observed and noted (Allen et al., 2004; Pavlov et al., 2004).

### 2.10 Quality control

Strict quality control was maintained to obtain reliable microbiological results. Instruments were calibrated before used. The quality of each agar plate was checked by incubating one plate of each batch in the incubator. To obtain reliable results a sample was processed three times. ATCC strains were used for quality control during antibiotic susceptibility testing. Strict aseptic conditions were maintained while executing the methodology.

### 2.11 Data analysis

Data analysis was done using Statistical Package for the Social Sciences (SPSS) version 19.

## 3 RESULTS

A total of 100 samples of bottled water were collected according to their local availability and popularity in the Kathmandu Valley. The samples were examined for microbiological parameters to assess the quality of bottled water. A total of 50 (25 monsoon and 25 postmonsoon) 20 L water bottles and 50 (25 monsoon and 25 postmonsoon) vessels of 1 L were examined in this study.

### 3.1 Parasite(s)

All 100 samples were subjected to iodine mount and wet mount tests. Parasite(s) were not detected. These results are presented in Table 1.

**TABLE 1:** Association between microbiological parameters (as per IBWA guideline), the volume of bottled water and season

Sn	Volume (L)	Microbiological parameters	Monsoon season		Postmonsoon season		Permissible value
			D	U (%)	D	U (%)	
1	1	Parasite(s)	X	0	0	0	<1
	20		X		0		
2	1	HPC	25	2	25	2	<25
	20		25	24	25	24	
3	1	Coliform count	2	2	2	2	<1
	20		24	24	24	24	
4	1	Fecal coliform count	0	0	0	0	<1
	20		24	24	10	10	
5	1	*Salmonella* spp	0	0	0	0	<1
	20		0		0		
6	1	*Vibrio* spp	0	0	0	0	<1
	20		0		0		
7	1	*Pseudomonas* spp	0	NA	0	NA	NA
	20		16		7		
8	1	*Pseudomonas aeruginosa*	0	NA	0	NA	NA
	20		16		7		
9	1	Coliphage	X	NA	0	NA	NA
	20		X		0		

Note

D, No. of water samples in which microorganisms were detected; NA, Not available in guideline; U, Unacceptability; X, Not detected.

### 3.2 Heterotrophic plate count

The range of HPC was found to be 15–1,300 cfu/ml. The HPC range for 20 L in monsoon and postmonsoon was found to be 24–200, 21–1,300 cfu/ml, respectively. And the HPC range for 1 L in monsoon and postmonsoon was found to be 15–30 and 16–28 cfu/ml, respectively. The results are presented in Table 1.

Chi‐square test shows that there is significant (*p* ≤ .05) association between HPC and volume of the vessel (i.e., 20 and 1 L), bottle type (i.e., unscratched and undented and scratched and/or dented); and there is no significant (*p* > .05) association between HPC and season (i.e., monsoon and postmonsoon). Pearson's correlation implies that there is a significant strong positive association between HPC and coliform count (*r* = 1.00), HPC and fecal coliform count (*r* = .690) and a significant negative association with HPC and *Pseudomonas* count (*r* = −.525), as seen in Table 2.

**TABLE 2:** Correlation between microbiological parameters

Parameters	HPC	Coliform count	Fecal coliform count	*Pseudomonas* count
HPC	1	1.00*	0.690*	−0.525**
Coliform count		1	0.690**	−0.525**
Fecal coliform count			1	−0.661*
*Pseudomonas* count				1

* Correlation is significant at the 0.05 level (2‐tail).

** Correlation is significant at the 0.01 level (2‐tail).

### 3.3 Total coliform count

Out of 100 samples, 48 (48%) samples were found to be within the guideline value of IBWA. Only 1 (1%) and 1 (1%) sample of 20 L bottled water were free from coliform, which was within guideline value IBWA in monsoon and postmonsoon, respectively. And only 23 (23%) and 23 (23%) samples of 1 L of bottled water were free from coliform, which was within guideline value IBWA in monsoon and postmonsoon, respectively. Based on the volume of bottled water, only 2 (2%) samples of 20 L and 46 (46%) samples of 1 L were acceptable as per IBWA guideline. These results are presented in Table 1.

Chi‐square test shows that there is a significant association (*p* ≤ .05) between total coliform and volume of the vessel (i.e., 20 and 1 L), bottle type (i.e., unscratched and undented and scratched and/or dented), HPC; and there is no significant (*p* > .05) association between total coliform and season (i.e., monsoon and postmonsoon). Pearson's correlation implies that there is a significant strong positive association between coliform count and fecal coliform count (*r* = .690) and a significant negative association between coliform count and *Pseudomonas* count (*r* = −.525), as seen in Table 2.

### 3.4 Fecal coliform

Among the 100 samples, 73 (73%) samples were found within the guideline value of the IBWA guideline. Only 8 (8%) and 15 (15%) samples of 20 L bottled water were free from coliform, which was acceptable as per guideline of IBWA in monsoon and postmonsoon, respectively. All 25 (25%) and 25 (25%) samples of 1 L of bottled water were free from coliform, which was acceptable as per guideline of IBWA in monsoon and postmonsoon, respectively. These results are presented in Table 1.

Chi‐square test shows that there is a significant association (*p* ≤ .05) between fecal coliform and volume of the vessel (i.e., 20 and 1 L), bottle type (i.e., unscratched and undented and scratched and/or dented), season (i.e., monsoon and postmonsoon), and total coliform. Pearson's correlation implies that there is a significant negative association between fecal coliform count and *Pseudomonas* count (*r* = −.661), as seen in Table 2.

### 3.5 *Pseudomonas* spp count

Out of 100 samples, 77 (77%) samples were found to be free from *Pseudomonas* spp. Only 9 (9%) and 18 (18%) sample of 20 L bottled water were free from *Pseudomonas* spp, in monsoon and postmonsoon, respectively. All 25 (25%) and 25 (25%) samples of 1 L of bottled water were free from *Pseudomonas* spp, in monsoon and postmonsoon, respectively. The plate incubated at 42°C implies and confirms that all isolates were *P. aeruginosa*. These results are presented in Table 1.

Chi‐square test shows that there is a significant association (*p* ≤ .05) between *Pseudomonas* spp and volume of the vessel (i.e., 20 and 1 L), bottle type (i.e., unscratched and undented and scratched and/or dented), HPC and total coliform; and there is no significant (*p* > .05) association between *Pseudomonas* spp and season (i.e., monsoon and postmonsoon).

### 3.6 Enrichment, isolation, and identification of pathogens

All 100 samples were subjected to enrichment and isolation. Pathogens (*Salmonella* spp and *Vibrio* spp) were not detected. These results are presented in Table 1.

### 3.7 Enrichment, isolation of coliphage

All 100 samples were filtered and the isolated *E. coli* was subjected to the overlay method. These results are presented in Table 1.

### 3.8 Antibiotic susceptibility test

#### 3.8.1 Antibiotic susceptibility pattern of *E. coli*

The isolated fecal coliform, *E. coli* were subjected to antibiotic susceptibility test. The isolates were susceptible to ofloxacin (100%), imipenem (100%), gentamicin (100%), chloramphenicol (70.59%); and resistant to ampicillin and chloramphenicol (29.41%). These results are presented in Table 3.

**TABLE 3:** Antibiotic susceptibility pattern of *Escherichia coli* isolated from fecal coliform

Sn	Antibiotic	Sensitive	Intermediate	Resistant
1	Ofloxacin	34 (100%)	‐	‐
2	Ampicillin	‐	‐	34 (100%)
3	Imipenem	34 (100%)	‐	‐
4	Gentamicin	34 (100%)	‐	‐
5	Chloramphenicol	21 (61.77%)	3 (8.82%)	10 (29.41%)

#### 3.8.2 Antibiotic susceptibility pattern of *E. aerogens*

The isolated fecal coliform, *Enterobacter aerogenes* were subjected to antibiotic susceptibility test. The isolates were susceptible to ofloxacin (100%), imipenem (85.3%), gentamicin (100%), chloramphenicol (70.06%); and resistant to ampicillin, imipenem (14.7%), and chloramphenicol (29.4%). These results are presented in Table 4.

**TABLE 4:** Antibiotic susceptibility pattern of *Enterobacter aerogenes*

Sn	Antibiotic	Sensitive	Intermediate	Resistant
1	Ofloxacin	34 (100%)	‐	‐
2	Ampicillin	‐	‐	34 (100%)
3	Imipenem	20 (58.83%)	9 (26.47%)	5 (14.7%)
4	Gentamicin	34 (100%)	‐	‐
5	Chloramphenicol	16 (47.06%)	8 (23.54%)	10 (29.4%)

#### 3.8.3 Antibiotic susceptibility pattern of *P. aeruginosa*

The isolated *P. aeruginosa* were subjected to antibiotic susceptibility test. The isolates were susceptible to polymyxin B (87%), ceftazidime (95.83%), imipenem (95.83%), gentamicin (83.33%), and piperacillin (50%); and resistant to polymyxin B (13%), ceftazidime (4.17%), imipenem (4.17%), gentamicin (16.67%) and piperacillin (50%). These results are presented in Table 5.

**TABLE 5:** Antibiotic susceptibility pattern of *Pseudomonas aeruginosa*

Sn	Antibiotic	Sensitive	Intermediate	Resistant
1	Polymyxin B	16 (69.6%)	4 (17.4%)	3 (13%)
2	Ceftazidime	20 (83.33%)	3 (12.5%)	1 (4.17%)
3	Imipenem	17 (70.83%)	6 (25%)	1 (4.17%)
4	Gentamicin	13 (54.16%)	7 (29.17%)	4 (16.67%)
5	Piperacillin	3 (12.5%)	9 (37.5%)	12 (50%)

#### 3.8.4 Threat assessment

The isolated fecal coliform (*E. coli*, *E. aerogenes)*, *P. aeruginosa* were subjected to a threat assessment test. Due to the production of virulence factor(s), 88.23% of the isolated *E. coli* produced β hemolytic colonies, while 11.77% didn't show hemolytic colonies. The *E. aerogenes* (100%) didn't give hemolytic colonies. The *P. aeruginosa* (100%) colonies gave β hemolytic colonies. These results are presented in Table 6.

**TABLE 6:** Threat possessed by the microorganism against erythrocytes

Sn	Microorganism	α Hemolysis	β hemolysis	γ hemolysis
1	*Escherichia coli*	‐	30 (88.23%)	4 (11.77)
2	*Enterobacter aerogenes*	‐	‐	34 (100%)
3	*Pseudomonas aeruginosa*	‐	24 (100%)	‐

## 4 DISCUSSION

In this study HPC for all the jar water samples showed 52 (52%) samples were found to be >25 cfu/ml. This result agreed with the results of Bedada et al. (2018) and Shahryari et al. (2020) who in their respective studies found that 51.4% and 61.8% samples were found to exceed the guideline value. In this study, the HPC count was in the range of 15–1,300 cfu/ml which agreed with the result of Bedada et al. (2018), Sala‐Comorera et al. (2019) and Shahryari et al. (2020) in which the range of HPC was found to be 1–99, 1–10,000 and 9,974–23,855 cfu/ml. HPC are a good indicator of the overall quality of production (WHO, 2017) and as recent studies have revealed that trace elements are present in ample amount in bottled water, the extremophiles may survive due to the optimum osmotic pressure (maintained by the microorganism in the bottled water) (Gautam, 2020; Gautam & Adhikari, 2018a; Gautam et al., 2021). Although HPC detects both pathogenic and nonpathogenic bacteria there is always a chance that higher HPC is associated with the presence of pathogens including coliforms (Gautam et al., 2021; Gautam, Rajbhanshi, et al., 2019). The source of contamination might be the during processing and handling (due to handling error) or natural source of water (Gautam & Adhikari, 2018b; Gautam et al., 2021). Bacteria can grow to the extent of affecting human health based on the length of storage condition of bottled water (Gautam et al., 2021). It has also been pointed out that the higher HPC is an indication of poor manufacturing practices involved in the processing of such water and must therefore be deemed to be unacceptable (Gautam et al., 2021). As per the result of chi‐square test, there was a significant (*p* ≤ .05) association between HPC and volume of vessel (i.e., 20 and 1 L), bottle type (i.e., unscratched and undented and scratched and/or dented); and there was no significant (*p* > .05) association between HPC and season (i.e., monsoon and postmonsoon). The decomposition of PET into formaldehyde and acetaldehyde is also brought by the heterotrophic bacteria in the presence of micronutrients in the bottled water (Gautam et al., 2021).

In total 52 (52%) samples were unacceptable according to IBWA guideline for total coliform. This result didn't agreed with the results of Bedada et al. (2018) and Shahryari et al. (2020) who in their respective studies found that 0.9% and 4.5% samples were found to exceed the guideline value; the difference in the result could be that the sample selected in the study of Bedada et al. (2018) and Shahryari et al. (2020) who selected only 1 L bottled water and didn't select the 20 L bottled water. Coliform organisms are representative of bacteria normally present in the intestinal tract of mammals including humans so are also known as indicator organisms (Gautam & Adhikari, 2018a, 2018b; Gautam, Aryal, et al., 2018; Gautam et al., 2021; Gautam, Rajbhanshi, et al., 2019). The presence of coliforms in 20 L bottled water indicates the potential presence of pathogens but also questions the standard guideline (Gautam et al., 2021). Coliforms have also been reported to have the capacity to form biofilms (Gautam & Adhikari, 2018b; Gautam et al., 2021). Chi‐square test revealed that there was a significant association (*p* ≤ .05) between total coliform and volume of the vessel (i.e., 20 and 1 L), bottle type (i.e., unscratched and undented and scratched and/or dented), HPC; and there was no significant (*p* > .05) association between total coliform and season (i.e., monsoon and postmonsoon). The result of this study was in agreement with the study of Gautam et al. (2021); highlighting the fact that when a microbial aspect is assessed the bottling, storage, transportation, and quality surveillance system are questionable. Contamination taking place during bottling processes can lead to bacterial growth especially if the water is stored under inappropriate conditions (Gautam et al., 2021). Pearson's correlation test revealed that there was a strong positive correlation between total coliform and HPC (*r* = 1.000, *p* < .01), this could be due to the majority of coliform among the heterotrophic bacteria present in bottled water.

Among the coliform isolates, 34% were found to be of fecal origin. This result did not agree with the result of Bedada et al. (2018) who in their respective studies found that 0.5% samples were found to exceed the guideline value, the reason could be due to exclusion of 20 L bottled water in their sample size. According to the IBWA & DFTQC guideline, *E. coli* and fecal coliform should not be detected in any water intended for consumption (DFTQC, 2016; IBWA, 2012). After being exposed to the environment it might have adapted survival mechanisms like integrating plasmid (Gautam, 2020; Gautam & Adhikari, 2018a, 2018b; Gautam, Aryal, et al., 2018; Gautam et al., 2021; Gautam, Dongol, et al., 2017; Gautam, Nepal, et al., 2019; Gautam, Pokhrel, et al., 2017; Gautam, Rajbhanshi, et al., 2019) and when consumed it might compete with the normal flora of our intestine (Gautam & Adhikari, 2018a, 2018b; Gautam, Aryal, et al., 2018; Gautam et al., 2021; Gautam, Rajbhanshi, et al., 2019). If it survives the competition then it might lead to morbidity and sometimes even death (Gautam & Adhikari, 2018b; Gautam et al., 2021). Pearson's correlation test revealed that there was a strong positive correlation between fecal coliform and HPC (*r* = .690, *p* < .01), this could be due to the majority of coliform among the heterotrophic bacteria present in bottled water. Pearson's correlation test revealed that there was a strong positive correlation between fecal coliform and total coliform (*r* = .690, *p* < .01), this could be due to majority of fecal coliform among the total coliform present in bottled water. Isolation of coliforms in bottled water reflect that morbidity, mortality, endemic, epidemic etc. are just around the corner and health of public should not be taken lightly or for granted (Gautam et al., 2021).

Among 100 samples, *Pseudomonas* spp was found in 23 (23%) of bottled water and all 23 of the bottled contained *P. aeruginosa*. This result agreed with the results of El Din (2019) and Molnár et al. (2020) who in their respective studies found that 32 and 3 samples were found to exceed. Biofilm of *Pseudomonas* spp has a specialized architecture ensuring well‐being and survival of cells that can develop on solid surfaces (Gautam & Adhikari, 2018a; Gautam, Rajbhanshi, et al., 2019). *Pseudomonas aeruginosa* will be able to grow at 42°C while other species of *Pseudomonas* cannot (Gautam, Aryal, et al., 2018). *Pseudomonas aeruginosa* can survive for several days in water lacking suspended particles as it can grow in very low nutrient (Gautam & Adhikari, 2018a; Gautam, Rajbhanshi, et al., 2019). *P. putida*, *P. monteilii*, *P. otitidis*, *P. mosselii*, *P. alcaligenes* and *P. pseudoalcaligenes* are unable to grow at 42°C and do not produce pyocyanin and are also nonhemolytic on blood agar (Holt et al., 1994). With the help of pili and outer membrane protein it adheres in the intestine and lead to bacteremia, septicemia and other clinical features (Gautam & Adhikari, 2018a; Gautam, Rajbhanshi, et al., 2019; Holt et al., 1994). Pearson's correlation test revealed that there was a strong negative correlation between *Pseudomonas* spp count and total coliform (*r* = −.525, *p* < .01), implying that *Pseudomonas* spp competes for their survival with total coliform.

Although bottled water was contaminated with coliform(s), pathogens like *Salmonella* spp and *Vibrio* spp were not detected in any water samples. This result agrees with the result of Bedada et al. (2018) where coliforms were detected in bottled water but *Salmonella* spp weren't detected. The acidic pH of bottled water might be the reason for the absence of *Vibrio* spp as it thrives in alkaline conditions and dies off in acidic conditions (Gautam, 2020; Gautam & Adhikari, 2018b; Gautam et al., 2021). *Salmonella* spp have a short life in water and they offer less resistance to adverse conditions (Gautam & Adhikari, 2018a; Gautam, Rajbhanshi, et al., 2019).

Coliphage wasn't isolated from all 100 samples, it could be due to unmatched unique receptors or due to undergoing the lysogenic cycle (Gautam & Adhikari, 2018b; Gautam, Aryal, et al., 2018). As no any pfu/ml was observed in any of the samples even after enrichment and introduction to *E. coli* (ATCC and strain isolated from waste water treatment plant, Guheswori) supported the fact that the groundwater utilized by the bottled water companies were free from coliphage contamination even in monsoon and postmonsoon season. In a recent study by Novello et al. (2020) bacteriophage have been used in seal caps of the bottled water to preserve the self‐life against *P. aeruginosa*.

Among the isolated strains of fecal coliforms, multidrug‐resistant *E. coli* and *E. aerogenes* were screened mostly resistant to ampicillin, chloramphenicol, and imipenem. Among the isolated strains of *P. aeruginosa*, multidrug‐resistant strains were screened mostly resistant to polymyxin B, imipenem, ceftazidime, gentamicin, and piperacillin.

After streaking the purified bacterial isolates on blood agar, 30 (88.23%) and 4 (11.77%) isolated of *E. coli* were found to be β and γ hemolytic; 34 (100%) isolates of *E. aerogenes* were found to be γ hemolytic and 24 (100%) isolates of *P. aeruginosa* were found to be β hemolytic. The result agreed with the study of Pavlov et al. (2004) where the isolated strains of *P. aeruginosa* produced β hemolytic colonies. For any microorganism to pose threat, it must contain a certain dose which is commonly known as infective dose (Holt et al., 1994; Waugh & Grant, 2014). If the dose is sufficient, then with the aid of virulence factors the etiological agent will evade human defense mechanisms eventually leading to morbidity (Allen et al., 2004; Waugh & Grant, 2014). Blood agar was used to detect the threat as it is physiologically equivalent to the human condition and hemolysis of blood can also be observed on blood agar due to the presence of virulence factors like hemolysin, colicin, aerobactin etc. (Holt et al., 1994; Waugh & Grant, 2014).

Opening bottled water companies without having a lab or technical supervision and/or both of the problems are due to ineffective surveillance by IBWA, DFTQC, WHO (Gautam, 2020; Gautam & Adhikari, 2018b; Gautam et al., 2021) etc.

## 5 CONCLUSION

The main conclusions are:

1. Out of 100 samples, 48% of samples were found to be contaminated with total coliform. *E. coli* was the predominant strain among the coliforms.
2. From the result of the study, it is clear that microbial quality of bottled water of 20 L bottled water and 1 L bottled water although being manufactured in the same season and of same company was found to vary as 1 L bottled water (except four samples) had no pathogen (including coliform) while the 20 L bottled water (except 48 samples) had no pathogen. This proves that the source of water is not contaminated but the water was contaminated during manufacturing process.
3. The problem of high HPC and coliform count is prominent across the globe.
4. Self‐cleaning bottle or self‐life extending techniques, both can be even achieved through the knowledge of CRISPR, genetic engineering, and nanotechnology.
5. Extremophile bacteria that survive on bottled water for a long time can incline to several survival mechanisms and may even solely survive on complex polymers like polyethylene terephthalate (PET). Due to microbial leaching, even carcinogens could also be present in the sealed bottled water.
